# Dengue in pregnancy and maternal mortality: a cohort analysis using routine data

**DOI:** 10.1038/s41598-018-28387-w

**Published:** 2018-07-02

**Authors:** Enny S. Paixao, Katie Harron, Oona Campbell, Maria Glória Teixeira, Maria da Conceição N. Costa, Mauricio L. Barreto, Laura C. Rodrigues

**Affiliations:** 1 London School of Hygiene and Tropical Medicine. Keppel St, Bloomsbury, London, WC1E 7HT United Kingdom; 2 Instituto de Saúde Coletiva. Rua Basílio da Gama, s/n.Canela, CEP 40110040 Salvador, Bahia Brazil; 3 Center of Data and Knowledge Integration for Health (CIDACS), Instituto Gonçalo Moniz, Fundação Oswaldo Cruz, CEP 41745-715 Salvador, Bahia Brazil

## Abstract

Dengue is a mosquito-borne disease with major public health importance due to its growing incidence and geographical spread. There is a lack of knowledge on its contribution to maternal death. We conducted a population-based cohort study to investigate the association between symptomatic dengue during pregnancy and deaths in Brazil from 2007 to 2012. We did this by linking routine records of confirmed dengue cases to records of deaths of women who had a live birth. Using the Firth method, we estimated odds ratios for maternal deaths associated with dengue during pregnancy. Dengue increased the risk of maternal death by 3 times (95%CI,1.5–5.8) and dengue haemorrhagic fever increased the risk of maternal death by 450 times (95%CI,186.9–1088.4) when compared to mortality of pregnant women without dengue. The increase in risk occurred mostly during acute dengue 71.5 (95%CI,32.8–155.8), compared with no dengue cases. This study showed an increased risk of adverse outcomes in pregnant women with dengue. Therefore in areas where dengue is circulating, the health of pregnant women should be not only a public health priority, but health professionals attending pregnant women with dengue should more closely observe these patients to be able to intervene in a timely way and avoid deaths.

## Introduction

Dengue is a mosquito borne disease with a major importance in the public health arena due to a growing incidence (30-fold rise in the past 50 years)^1^ and an expanding geographical range (endemic in more than 100 countries, mostly in South America and Southeast Asia and still spreading to new areas, including Europe)^2^. According to the World Health Organization (WHO), approximately half the world’s population is at risk^2^. However the burden of dengue during pregnancy on maternal ill-health is not well understood. Physiological changes that occur during pregnancy (such as hemodilution) can mask the thrombocytopenia, leucopoenia, or hemoconcentration associated with dengue, and common obstetric problems can cause haematological and hepatic issues, masking the disease. These may make it difficult to differentiate dengue haemorrhagic fever from common obstetric conditions, leading to misdiagnosis^3,4^.

Dengue during pregnancy has been associated with poor fetal and maternal outcomes. There is some evidence that the risk of severe dengue and of hospitalization due to dengue is higher among pregnant compared with non-pregnant women^5^ and a number of case series have reported maternal deaths, and other complications such as bleeding and iincreased caesarean section rates as being associated with dengue^6^, Two small cohorts comparing pregnant women exposed to dengue or in Brazil and Colombia to those not exposed found more maternal deaths among the dengue exposed group^7,8^.

Ourstudy we analysed a large population-based retrospective cohort to investigate these issues in greater detail, and with greater power to explore the association between symptomatic dengue during pregnancy, and maternal mortality.

## Methods

We conducted a population-based retrospective cohort study by linking routine records of all women notified and confirmed as having dengue, with all records of maternal deaths following delivery of a live birth, in Brazil from January 1, 2007 to December 31, 2012.

### Data sources

We extracted routinely collected data from three Brazilian databases.

1. The Live Births Information System (Sistema de Informação sobre Nascimentos,m; SINASC), which contains records of all live births in Brazil; these data come from birth registration, a legal document completed by the health provider who assisted the delivery. SINASC includes information on the women who gave birth, includingas the woman’s name, place of residence, age, marital status, education; the pregnancy (length of gestation, type of delivery); and the newborn (weight at birth, the presence of birth anomalies and gestational age at birth)^9^. The data completeness and coverage are very high, with more than 90% completeness for most variables and capturing 97% of Brazilian registered births^10,11^.
2. Mortality Information System (Sistema de Informação sobre Mortalidade; SIM), which contains records of all deaths in Brazil, incluing fetal deaths; these data come from the Death Certificate, a required legal document^12^. We retained all deaths of women coded under obstetric causes of death by ICD-10, the “O” group. The proportion of records with missing data varied by variable (e.g. maternal education was missing in 23% of records). Although stillbirth was not an outcome of this study, we retained all fetal deaths to link with maternal deaths as described below.
3. Notifiable Diseases Information System (Sistema de Informação de Agravos de Notificação; SINAN), which contains records on notifiable diseases. The dengue notification system includes information on the individual such as name, place of residence, age, sex, and years of education, on the disease, such as symptoms, laboratory tests, and disease severity^13^. For linkage, <0.05% records were excluded because of missing name, and around 5% of dengue cases did not have a final classification of severity. Laboratory confirmation is not required to confirm dengue in Brazil. According to the Ministry of Health, around 30% of dengue cases were laboratory confirmed at the time^14^. During this period, dengue was the main (and sometimes the only) vector-borne disease circulating in Brazil, as yellow fever and malaria occurred in restricted areas, and Zika and Chikungunya were not circulating until 2014.

### Procedures

Maternal death records that linked with a stillbirth, a pregnancy with an abortive outcome, or that failed to link were excluded from analysis. Although we linked maternal deaths to livebirths and stillbirths, we only used the maternal deaths linked with live births in this study, because the comparison group was live births that occurred in Brazil during the study period.

Ethical approval was obtained from The Federal University of Bahia, Salvador, Brazil (CAAE: 26797814.7.0000.5030) and from The London School of Hygiene and Tropical Medicine (Ethics Ref:10269).

### Outcomes

**Figure 1:**
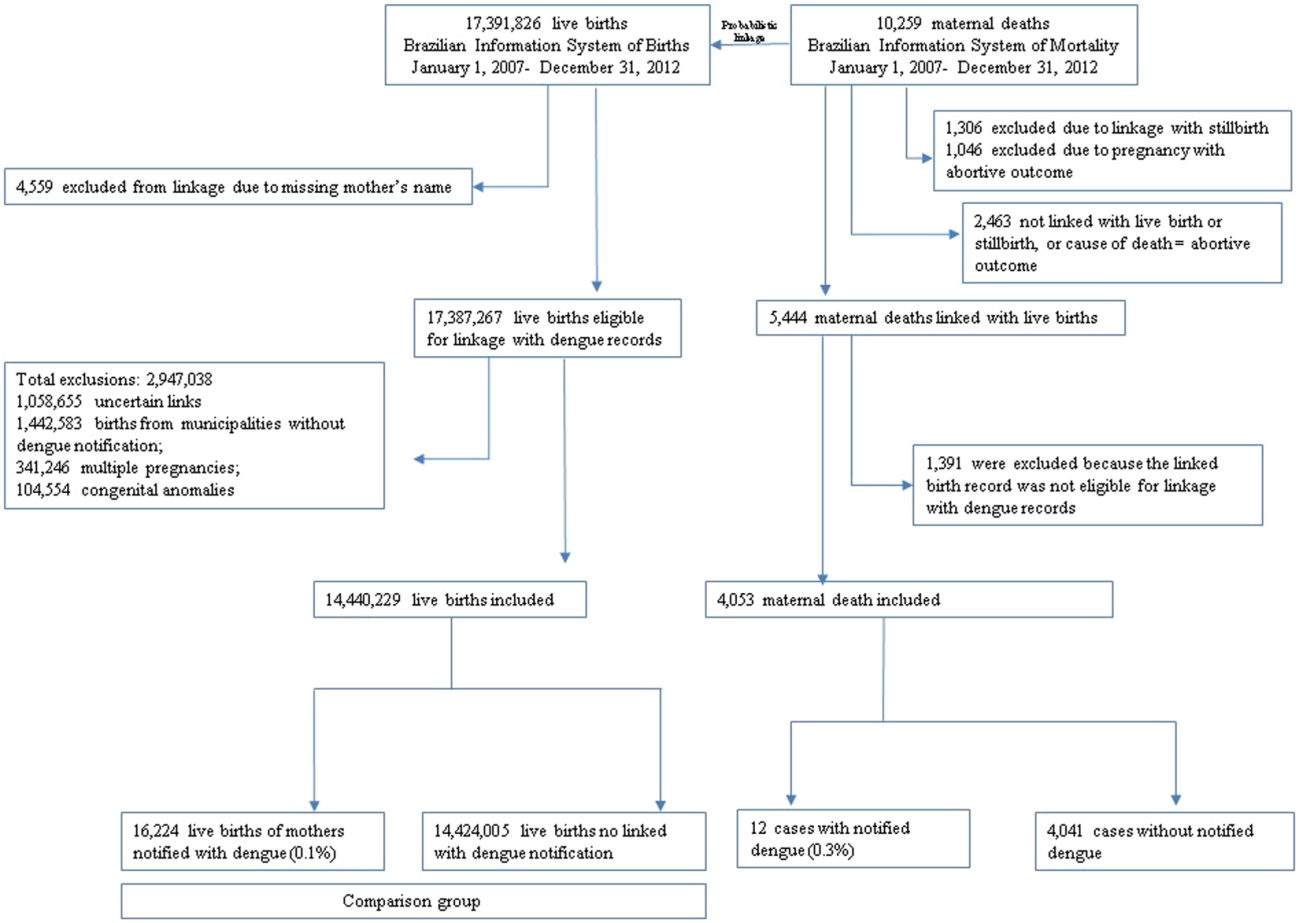
Flowchat with the linkage information Brazil, 2007–2012.

We included female deaths with an obstetric code in ICD-10 recorded as the cause of death. The comparison group in our study was women who had a live birth in the same period in Brazil who were not excluded during the linkage process described below (Fig. 1).

### Exposure

The exposure under study was being a confirmed case of dengue notified during a pregnancy that resulted in a live birth. In Brazil, dengue confirmation can be based on clinical/epidemiological criteria, namely presence of clinical symptoms of dengue in the same area and time as other confirmed cases of dengue, or on clinical/laboratory criteria, namely the presence of clinical symptoms and a positive IgM ELISA result, viral RNA detection via PCR, NS1 viral antigen detection, or positive viral culture^15^. We considered maternal deaths to be “exposed” if the woman’s records linked with a live birth that was previously linked to a confirmed dengue case. We defined “dengue during pregnancy” as all confirmed cases of dengue (clinical/epidemiological or clinical/laboratorial). Laboratory confirmed cases were referred to as “dengue during pregnancy, laboratory confirmed”. We used the same three clinical categories of classification as used by the Brazilian Ministry of Health at the time of the study: “dengue fever” (a self-limiting fever, with a severe headache, pain behind the eyes, muscle and joint pain, and rash), “complicated dengue”, and “dengue haemorrhagic fever/dengue shock syndrome”. Complicated dengue is a Brazilian definition of severe cases of dengue that do not meet the WHO criteria for dengue haemorrhagic fever (i.e. fever, haemorrhagic evidence, thrombocytopenia and evidence of plasma leakage) but that cannot be classified as mild self-limited disease due to their severity. Complicated dengue is used when a probable case of dengue presents with one of the following: severe changes in the nervous system, cardiorespiratory dysfunction, insufficient hepatic function, gastrointestinal bleeding, cavity spills, or thrombocytopenia equal or less than 50,000/mm^3^, leucometry less than 1000/mm^3 ^^15^.

### Linkage process

We conducted the linkage in two steps. First, we probabilistically linked records of dengue notifications (SINAN) with records of live births (SINASC) and stillbirths (SIM) to identify those women who had dengue during pregnancy and who gave birth. For simplicity, we refer to these as mothers even if the woman had a stillbirth. To link, we used the name of the mother, two sources of age, and place of residence of the mother at the time of delivery or notification. We excluded records with missing names (Fig. 1). Match weight calculations were based on the Fellegi-Sunter method^16^.

We then linked this composite births file^17^ (live births/stillbirth linked with maternal dengue status) with maternal death records using name, age, place of residence of the mother at time of delivery or death, and the time between the birth of the new-born and death of the mother. The procedures and evaluation of the matching process are the subject of a separate paper. However, in brief, we expected that after excluding pregnancies with abortive outcomes (10% of maternal mortality notifications), the large majority of the maternal deaths would have linked with a live birth or stillbirth. Although we did not use the maternal deaths linked to stillbirth for the analyses of this paper, it is important to include this stage in the linkage process section to present our measure of error in a comprehensive way. Women coded as having an abortive outcome that linked to a live birth record were assumed to be a false match. Maternal deaths linking to a live birth and a stillbirth simultaneously were also classified as false matches, unless they were multiple births.

We linked 6593 maternal deaths to the composite births file, of which 65 were identified as false matches. This gave a positive predictive value (PPV) of 6528/6593 = 99%. Of 9213 maternal deaths without an abortive outcome, we were unable to link 2,675, giving a sensitivity of 6,528/9213 = 71%. Mothers with more than 7 years of education and self-declared as Caucasian were more likely to link with the composite live birth stillbirth file.

We further evaluated potential linkage errors using dengue information obtained from the first linkage (between dengue and live births). We compared the maternal deaths classified as having dengue as a cause of death that linked with a live birth or stillbirth, with maternal deaths where dengue was coded as an underlying case of death-ICD-10 but that had e not linked. There was no difference in socio-demographic characteristics between these two groups, suggesting that although we did not capture all of the matches in our linkage, there was no selection bias associated with the exposure in our linked cohort.

### Statistical analysis

Using a Chi square test, we compared the characteristics of women by dengue status. We estimated the crude and adjusted odds ratio using the Firth method^18^ (to reduce the small sample bias in maximum likelihood estimation) as we were analysing rare events, controlling for age, education and mode of delivery. For a sensitivity analysis of the validity of clinical/epidemiologic diagnosis, we repeated the analyses using only laboratory confirmed dengue. We investigated the effect of dengue severity (mild dengue, dengue with complications and dengue haemorrhagic fever) and time between disease onset and maternal deaths, since in general, dengue is an acute disease with rapid recovery. The time between disease onset and the outcome was calculated using the date of the disease onset (information available in SINAN) and the date when the outcome occurred (date of maternal death); we categorized this difference as being less than or equal to ten days or greater than 10 days.

### Data availability

The data that support our study findings are available from Brazilian Ministry of Health but restrictions apply to the availability of these data, which were used under license for the current study, and so are not publicly available. Data are however available from the Brazilian Ministry of Health upon reasonable request.

## Results

The Brazilian Information System recorded 10,259 maternal deaths from 2007–2012. After exclusions, 4,053 maternal deaths in women with live births were included in our study population, and 12 (0.3%) had a positive dengue status (Fig. 1). The live birth system recorded 17,391,826 live births from 2007–2012. After exclusions, 14,440,229 women with live births were included in the study, and 16,224 (0.1%) had a dengue notification record.

The cohort characteristics by dengue status are described in Table 1. Compared with women without dengue, women with dengue were more likely to have more years of formal education, and to have a caesarean section as a mode of delivery.

**Table 1:** Maternal characteristics and delivery details in relation to dengue status, Brazil, 2006–2012.

Characteristics	Notified with confirmed dengue in pregnancy n (%)	Without dengue notification n (%)	p value
Age of the mother			
<20	4,127 (25.4)	3,738,246 (25.9)	<0.001
20–35	11,011 (67.8)	9,560,187 (66.3)	
>35	1,097 (6.8)	1,129,318 (7.8)	
Missing	3 (0.0)	1,488 (0.0)	
Maternal education			
Less than 3 years	946 (5.9)	1,099,923 (7.8)	<0.001
4–7 years	4,239 (26.7)	3,867,130 (27.4)	
More than 8 years	10,710 (67.4)	9,153,016 (64.8)	
Missing	343 (2.1)	309,170 (2.1)	
Delivery			
Vaginal	7,599 (46.9)	7,117,183 (49.4)	<0.001
C-section	8,618 (53.1)	7,286,995 (50.6)	
Missing	21 (0.1)	25,061 (0.2)	
Maternal Mortality			
Yes	12 (0.1)	4,041 (0.03)	<0.001
No	16,224 (99.9)	14,424,005 (99.97)	

Dengue during pregnancy tripled the risk of maternal death from 0.1% (16,224/14,440,229) to 0.3% (12/4,053), OR 3.0 (95% IC 1.3–5.8) (Table 2). The proportion of dengue cases that were laboratory confirmed was much higher among maternal deaths (9/12 = 75%) than among surviving mothers who had live births (5309/16224 = 33%). Restricting analyses to laboratory-confirmed cases showed that dengue increased the odds of maternal death by almost eight fold (7.8 95% CI 3.1–15.4).

**Table 2:** Number of cases of dengue during pregnancy and Odds ratio (crude and adjusted) for the association between dengue during pregnancy and maternal death. Brazil, 2007–2012.

Outcome	All dengue cases		Laboratory confirmed dengue cases	
	Crude Risk Ratio (95% Confidence Interval)	Adjusted Risk Ratio (95% Confidence Interval)	Crude Risk Ratio (95% Confidence Interval)	Adjusted Risk Ratio (95% confidence Interval)
Maternal Mortality^§^				
Frequency	12		9	
Overall (OR)	2.7 (1.6–4.8)	3.0 (1.5–5.8)	6.4 (3.4–12.1)	7.8 (3.8–15.9)

Adjusted for age, education and mode of delivery.

Complicated dengue increased the odds of maternal deaths by 27 fold (95% CI 5.5–136.3), compared with those with no dengue. However, this number was much higher among the women that developed dengue haemorrhagic fever, for whom the increase in risk of maternal death was 451 fold (95% CI 186–1088) (Table 3).

**Table 3:** Number of cases of dengue during pregnancy (N) and Odds Ratio (crude and adjusted) for the association between dengue during pregnancy by severity of disease and maternal death. Brazil, 2007–2012.

Outcome	Mild dengue Odds Ratio (95% Confidence Interval)	Complicated dengue Odds Ratio (95% Confidence Interval)	Haemorrhagic fever Odds Ratio (95% Confidence Interval)
Maternal Mortality^§^			
Frequency	3	2	5
Crude	0.8 (0.3–2.3)	28.7 (8.2–99.7)	274.5 (115.3–653.8)
Adjusted	0.95 (0.3–3.3)	27.3 (5.5–136.3)	451 (186.9–1088.4)

Adjusted for age, education and mode of delivery.

The risk of maternal death among pregnant women with dengue depended on the time between first symptoms of dengue and the date of death; the deaths occurred mainly during acute disease, (i.e. within10 days of the disease’s onset (Table 4).

**Table 4:** Number of cases with dengue during pregnancy (N) and Odds Ratio for the association between dengue during pregnancy by timing of disease and maternal death. Brazil, 2007–2012.

Outcome	Odds Ratio and frequency of outcomes within 10 days of disease onset (95% Confidence Interval)	Odds Ratio and frequency of outcomes after 10 days from disease onset (95% Confidence Interval)
Maternal Mortality^§^		
Frequency	8	4
Crude	62 (31.6–122.6)	1.0 (0.4–2.6)
Adjusted	71.5 (32.8–155.8)	0.9 (0.3–3.1)

Pre-eclampsia or eclampsia was the registered cause of death in 25% of the patients with dengue; in the group without dengue, but who had a live birth, these causes were responsible for 19% of all deaths.

## Discussion

In this retrospective cohort, we found that pregnant women with symptomatic dengue had a significantly higher risk of maternal death compared to pregnant women without dengue, and this risk was considerably higher when dengue was severe, complicated, or dengue haemorrhagic fever. The proportion of laboratory confirmed cases among women who died was much higher than among the comparison group, and the effect of dengue on death occurred primarily during the acute disease, in the first ten days after disease onset.

The results of this study are consistent with the literature that shows a higher maternal mortality ratio associated with dengue, mainly among those who developed severe dengue. In a Brazilian cohort in Rio Branco, the maternal mortality ratio in the dengue exposed group was 13 times the mean maternal mortality ratio of the area^8^ as opposed to our finding of 3 times higher. The percentage of maternal deaths among pregnant women with dengue in the case series studies varied from 6.6% in Sri Lanka^19^ to 21.7% in the South Sudan^6^, whereas we see 0.07%. This may be because we included women with less severe disease or excluded women with stillbirth or other abortive outcomes. Our low sensitivity (71%) to detect dengue may also have palyed a role. Among women with severe disease and a live birth, the mortality rate was 0.12%.

The mechanism for the association between dengue and maternal deaths is not clear: one possible explanation is that the clinical aspects of the disease may be different during pregnancy in a way that increases the susceptibility to dengue haemorrhagic fever. In Brazil two studies showed that pregnant women with dengue were 3 times more likely to develop severe dengue or die due to this viruse than non-pregnant women^5,20^. The last study included the same dataset used in our study; however the selection of the exposure (dengue during pregnancy) used two different methodology, in this one we performed a linkage and Nacimento *et al*. (2017)^20^ used the information available in the dengue notification form. Because of that the numbers are not the same but the results were similar. Another hypothesis is that physiological changes during pregnancy such as hemoconcentration and the difficultly in distinguishing between severe dengue and common obstetric conditions may have led to misdiagnosis and a delay in disease treatment, that in turn can progress to hypovolemic shock and death. A meta-analysis of the effect of infection on pregnancy showed that viral infections can increase the risk of pre-eclampsia, although the authors do not mention dengue. It is possible that dengue virus leads to the same etiologic pathway of inflammatory modifications of placental tissues^21^. Pre-eclampsia was the cause of death in 25% of the patients with dengue, compared with 19% in the comparison group.

There is growing evidence that different infectious diseases during pregnancy can be associated with adverse outcomes. The evidence is still fragmented and the effect of different infections appear to vary with the pathogen involved and the severity of maternal disease. According to a meta-analysis, pregnant women with active tuberculosis tended to be more liked to suffer a maternal death although this was not significant (OR:4, 95% CI 0.65–25.2)^22^, and pregnant women with measles had a 9-fold increased risk of maternal death^23^.

The potential study limitations are associated with the linkage^17^. We undertook a rigorous validation process that showed that despite the low sensitivity, that kept us from making statements about the prevalence of dengue during pregnancy, it is unlikely that the linkage process introduced bias in the measure of association, because missed and false matches occurred randomly in the population. The maternal death linkage analysis comparing dengue cases that the algorithm was capable of capturing, with those that it could not, but which had recorded maternal death as cause of death, showed no difference between the two groups. Tthis result should be interpreted cautiously because it had limited power, due to the sample size.

There are some other limitations associated with the use of secondary data: we only had a limited number of possible confounders to analyze. Although we adjusted for these, unknown confounders, such as maternal co-morbidities or quality of care, may have contributed to the association between severe dengue and maternal deaths. Another limitation is the exclusion of miscarriages and stillbirths; dengue has been associated with an increased risk of these two conditions, and by excluding of them in our analyses, we may be underestimating the risk.

In summary, this study suggests a marked increase in the risk of maternal deaths in women with dengue during pregnancy. The health of pregnant women is a public health priority, but in places where dengue is circulating and the health professionals attending pregnant women with dengue should observe them more closely to be able to intervene in a timely way, and avoid death. We recommend further research in different settings to confirm our results and studies of negative fetal and maternal outcomes in other vector-borne diseases.
